# Comparative effectiveness of minimally invasive endoscopic discectomy versus conventional surgical techniques for lumbar disc herniation: a systematic review and meta-analysis

**DOI:** 10.1097/MS9.0000000000003689

**Published:** 2025-08-11

**Authors:** Tirath Patel, Muhammad Farhan, Dena Nashaat Hamza, Maral Daneshpazhouh, Odai Al Nahar, Ahmed El Serafy, Amro Gamal Mohamed Hasan, Lamia Bnaian, Mohamed Hameed Ali, Abdulaziz Sobhi

**Affiliations:** a Department of Surgery, Trinity Medical Sciences University School of Medicine, Ratho Mill, Kingstown, Saint Vincent and the Grenadines; b College of Medicine, Ajman University, Ajman, United Arab Emirates

**Keywords:** endoscopic discectomy, lumbar disc herniation, meta-analysis, minimally invasive surgery, systematic review

## Abstract

**Background:**

While traditional open discectomy is the standard surgical treatment for lumbar disc herniation (LDH), minimally invasive endoscopic techniques have emerged as potential alternatives. This systematic review and meta-analysis evaluated the comparative effectiveness of endoscopic discectomy (ED) and conventional surgical techniques (CT).

**Methods:**

A comprehensive search of the PubMed, Scopus, Cochrane Library, and Web of Science databases was conducted through January 2025. Randomized controlled trials and high-quality observational studies comparing ED and CT for LDH were included. Primary outcomes included Visual Analog Scale scores for back and leg pain (VAS-Back and VAS-Leg) and the Oswestry Disability Index (ODI). The secondary outcomes included complication rates, reoperation rates, and length of hospital stay.

**Results:**

Fourteen studies (1795 participants) met the inclusion criteria. Meta-analysis revealed no significant differences between ED and CT in terms of VAS-Back [standardized mean difference (SMD): 0.02; 95% CI: −0.15 to 0.19], VAS-Leg (SMD: −0.02; 95% CI: −0.19 to 0.16), or ODI scores (SMD: −0.09; 95% CI: −0.27 to 0.08). The complication rates (RR: 0.85; 95% CI: 0.55–1.31) and reoperation rates (RR: 1.00; 95% CI: 0.75–1.33) were comparable between the groups. ED was associated with a significantly shorter hospital stay (SMD: −2.40; 95% CI: −4.31 to −0.49).

**Conclusion:**

Minimally invasive ED is comparable to CTs in terms of pain relief, functional improvement, and safety profiles, while potentially offering the advantage of a shorter hospital stay. These findings support the viability of endoscopic approaches as effective alternatives to traditional surgery for LDH, although the surgical technique selection should be individualized based on patient characteristics, surgeon expertise, and available resources.

## Introduction

Lumbar disc herniation (LDH) is a prevalent spinal condition marked by the displacement of the nucleus pulposus material through the annulus fibrosus, leading to significant low back pain, radicular symptoms, and neurological deficits^[1]^. The mechanical compression of nerve roots, coupled with the inflammatory response, not only diminishes the quality of life but also imposes a substantial burden on healthcare systems^[2]^. LDH is one of the most common causes of chronic low back pain and radiculopathy worldwide, with an estimated prevalence of about 14–20% in the adult population. Approximately two-thirds of adults experience low back pain in their lifetime, and up to 85% of LDH patients report sciatica due to nerve-root compression. LDH is a leading cause of disability and imposes a heavy socio-economic burden globally^[3]^. This high burden of disease motivates the need for effective yet safe surgical treatments.

**HIGHLIGHTS**

- Endoscopic and conventional discectomy yield similar pain relief outcomes.
- Endoscopic discectomy shortens hospital stays compared to conventional methods.
- Both techniques show comparable safety and complication rates.
- Endoscopic approaches enhance recovery with less tissue disruption.
- Technique choice depends on patient needs, surgeon expertise, and resources.

While conservative treatments such as physical therapy and analgesics are often the first line of management, many patients require surgical intervention when these measures fail or in the presence of severe neurological impairment^[4,5]^. Various surgical techniques have been developed to address LDH, each differing in methodology, invasiveness, and outcome. Traditional open discectomy, which allows direct visualization and removal of herniated disc material, has long been the gold standard for immediate relief of symptoms^[6,7]^. However, the advent of minimally invasive techniques, such as percutaneous transforaminal endoscopic discectomy (PTED) and tubular minimally invasive discectomy, has revolutionized the surgical landscape^[8]^. These techniques require smaller incisions, reduced tissue disruption, and potentially shorter recovery times^[9]^. However, their efficacy relative to conventional methods remains a topic of debate owing to variations in surgical expertise, patient selection, and long-term outcomes^[10]^.

Recent comparative analyses suggest that, while standard open discectomy often provides rapid symptom alleviation, minimally invasive approaches may result in quicker recovery, lower postoperative discomfort, and reduced complication rates^[11]^. Despite these advantages, the effectiveness of these minimally invasive techniques can be influenced by the surgeon’s learning curve, patient characteristics, and specific clinical scenario^[12]^. Additionally, long-term outcomes, such as recurrence rates, sustained pain relief, and quality of life improvements, present mixed evidence, fueling ongoing discussions on the optimal surgical approach for LDH^[13]^.

This meta-analysis synthesizes evidence from multiple randomized controlled trials (RCTs) and high-quality observational studies to evaluate the postoperative outcomes, recovery times, complication rates, and long-term efficacy of various surgical interventions for LDH. This study aimed to elucidate differences in pain relief, functional recovery, and complication profiles by systematically comparing standard open discectomy with minimally invasive techniques, such as PTED and tubular discectomy. The overarching goal is to provide clinicians and patients with evidence-based insights that can guide surgical decision-making and tailor treatment strategies to individual patient needs and ultimately enhance the quality of care for those afflicted by LDH.

Previous meta-analyses comparing ED and CT have reported inconsistent findings regarding clinical outcomes. Some studies suggest that ED offers advantages such as reduced blood loss and shorter hospital stays, while others find no significant differences in postoperative pain or functional recovery between the two techniques^[14–16]^. These discrepancies may stem from variations in study designs, patient populations, and surgical techniques. To address these inconsistencies, our study incorporates both RCTs and observational studies, providing a more comprehensive analysis of current evidence. Additionally, we include recent studies and assess a broader range of outcomes, such as reoperation rates and length of hospital stay, to offer a more nuanced comparison between ED and CT.

## Methods

This systematic review and meta-analysis were conducted in accordance with the Preferred Reporting Items for Systematic Reviews and Meta-Analyses (PRISMA) guidelines^[17]^ and followed the recommendations of the Cochrane Handbook for Systematic Reviews of Interventions^[18]^. The review also followed the AMSTAR guidelines. This work has been reported in line with the AMSTAR (Assessing the Methodological Quality of Systematic Reviews) guidelines^[19]^.

### Eligibility criteria

We included studies that met the following criteria: patients undergoing surgical interventions for LDH or spinal stenosis; comparisons between minimally invasive endoscopic discectomy (ED) techniques and conventional open or minimally invasive surgical techniques (CT), including procedures such as PTED and microendoscopic discectomy (MED); and reporting at least one of the following outcomes: Visual Analog Scale (VAS) scores for back and leg pain (VAS-Back and VAS-Leg), Oswestry Disability Index (ODI), complication rates, reoperation rates, and length of hospital stay. Eligible study designs included RCTs and high-quality observational studies (nonrandomized comparative studies). Studies were excluded if they were case reports, reviews, animal studies, non-full-text publications, and non-English articles, or if they did not provide sufficient data for extraction. We included both RCTs and observational studies in our meta-analysis to capture a comprehensive range of clinical evidence. This approach acknowledges the limited number of RCTs available on this topic and the valuable insights that well-conducted observational studies can provide regarding real-world clinical practices and outcomes

### Literature search strategy

A comprehensive literature search was conducted across four electronic databases, PubMed, Scopus, Cochrane Library, and Web of Science, from inception until January 2025. The search strategy combined terms related to “endoscopic discectomy,” “minimally invasive spine surgery,” “lumbar disc herniation,” “microendoscopic discectomy,” “PTED,” “MED,” and relevant synonyms. No language restrictions were applied. The full search strategy for each database is provided in Supplemental Digital Content Table 1, available at: http://links.lww.com/MS9/A909; http://links.lww.com/MS9/A910; http://links.lww.com/MS9/A911.

### Study selection

All identified records were exported to the EndNote reference management software^[20]^ for the organization. Duplicates were removed using EndNote automated tools, followed by manual verification. Two independent reviewers screened the titles and abstracts against the eligibility criteria using Rayyan, a web-based systematic review screening tool^[21]^. Potentially relevant articles were retrieved for full-text review. Discrepancies between the reviewers were resolved through discussion or consultation with a third reviewer.

### Data extraction

Two reviewers independently extracted data from the included studies using a standardized data extraction form. Extracted data included study characteristics (author, year, country, study design, sample size, and follow-up duration), patient characteristics (age, sex, affected spinal level, and baseline clinical status), intervention details (ED techniques and CT, anesthesia type, and operative times), and outcomes (VAS-Back, VAS-Leg, ODI scores at various follow-up points, complication rates, reoperation rates, and hospital stay duration). Any discrepancies in the data extraction were resolved by consensus.

### Quality assessment

The methodological quality of RCTs was assessed independently by two reviewers using the Cochrane Risk of Bias 2 (ROB 2) tool^[22]^, which evaluates bias across domains, such as randomization, allocation concealment, blinding, incomplete outcome data, and selective reporting. A single nonrandomized study was assessed using the Newcastle–Ottawa Scale (NOS)^[23]^ to examine selection, comparability, and outcome assessment. Disagreements were resolved by discussion or by a third reviewer.

### Data synthesis and statistical analysis

Meta-analyses were performed using R version 4.4.2 with relevant statistical packages for meta-analysis (*meta* and *metafor*). For continuous outcomes (VAS scores, ODI scores, and hospital stay duration), the standardized mean difference (SMD) with 95% confidence intervals (CIs) was calculated. Risk ratios (RRs) with 95% CIs were used for dichotomous outcomes (complication and reoperation rates). A random-effects model was chosen *a priori* because of the expected clinical and methodological heterogeneity among studies.

Statistical heterogeneity across studies was assessed using the chi-squared (*χ*^2^) test and quantified using the *I*^2^ statistic and *τ*^2^ values. Heterogeneity was interpreted as low (*I*^2^ < 25%), moderate (*I*^2^ 25–75%), or high (*I*^2^ > 75%). When substantial heterogeneity was detected, potential sources were explored using sensitivity analyses, subgroup analyses, or outlier studies.

Preplanned sensitivity analyses were conducted to assess the robustness of the pooled estimates by excluding studies with a high risk of bias, specific outliers, or differing methodologies. Changes in the effect size and heterogeneity measures were observed to ensure the consistency of the results.

Publication bias was evaluated by visual inspection of funnel plots for each outcome. Egger’s regression test was employed to statistically assess funnel plot asymmetry. The trim-and-fill method was used to determine the potential impact of the unpublished studies on the results.

## Results

### Study identification and selection

**Figure 1.**
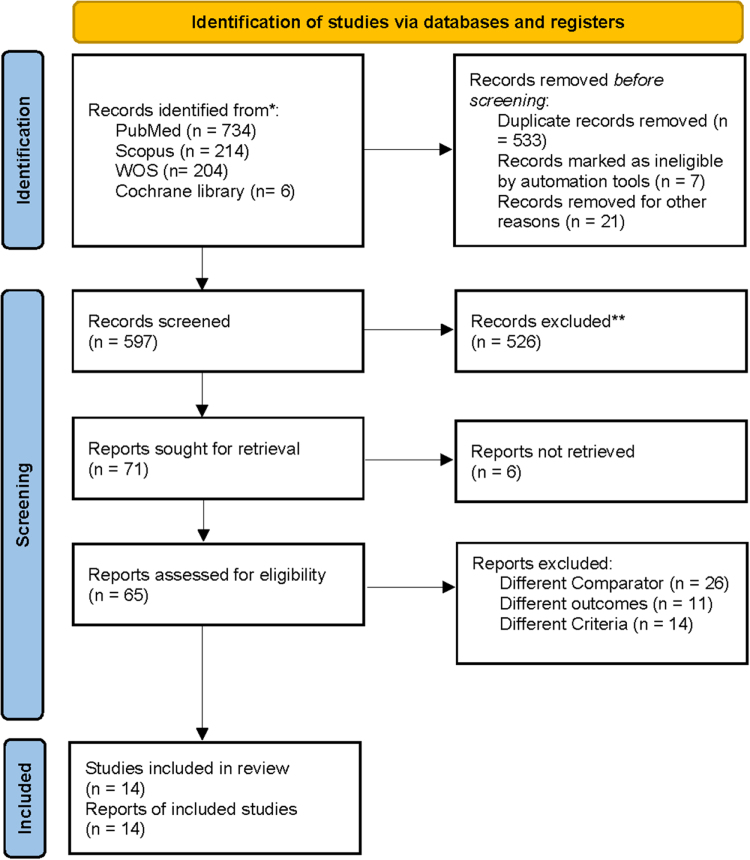
PRISMA flow diagram showing study selection process.

Our comprehensive literature search, conducted in accordance with the PRISMA guidelines, initially identified 1158 potentially relevant citations across electronic databases (PubMed, *n* = 734; Scopus, *n* = 214; Cochrane Library, *n* = 6; and Web of Science, *n* = 204). After removing 533 duplicates through automated and manual screening, 597 unique citations remained for the title and abstract screening. Of these, 532 articles were excluded based on predefined criteria. The remaining 65 articles underwent full-text evaluation, resulting in 14 studies^[24–36]^ that met all inclusion criteria for qualitative and quantitative synthesis (Fig. 1).

### Study and patient characteristics

The 14 included studies comprised 13 RCTs and one retrospective comparative study, encompassing 1795 participants. Studies were predominantly conducted in Asia (eight studies), followed by Europe (four studies) and South America (two studies). The sample sizes ranged from 20 to 325 participants, with follow-up periods ranging from 6 months to 5 years. The included studies compared various surgical techniques, with PTED and MED being the most common (Table 1).

**Table 1:** Summary of included studies

Study	Country	Study type	Sample size	Follow-up duration	Intervention	Comparator	Eligibility criteria	Key findings
Chen 2018	China	RCT	153	1 year	PTED	MED	Persistent radiculopathy, imaging-confirmed LDH, age 18–65, no urgent surgical needs	PTED is not superior to MED over 1 year. Comparable safety and efficacy.
Chen 2022	China	RCT	241	5 years	PTED	MED	Persistent radiculopathy, imaging-confirmed LDH, age 18–65, no urgent surgical needs	PTED and MED have comparable outcomes over 5 years. No significant long-term differences.
Chen 2019	China	RCT	241	2 years	PTED	MED	Persistent radiculopathy, imaging-confirmed LDH, age 18–65, no urgent surgical needs	PTED is not superior to MED over 2 years. Faster recovery but higher cost.
Cristante 2016	Brazil	RCT	40	1 year	Hydrodiscectomy	Open microdiscectomy	Disc protrusion/small herniation, one level, no neurological deficits, conservative treatment failure	Hydrodiscectomy is as effective as open microdiscectomy in reducing pain, with similar complication rates.
Gibson 2016	UK	RCT	143	2 years	TED	Microdiscectomy	Single-level lumbar prolapse, radiculopathy, age 25–70, failed conservative management	TED showed faster recovery and reduced hospital stay but had higher revision rates.
Jing 2021	China	Retrospective comparative study	62	1 year	PTED	MED	ULDH, single segment, failed conservative treatment, no segmental instability	PTED demonstrated smaller incisions, faster recovery, and better short-term outcomes.
Kong 2018	China	RCT	40	2 years	PELD	ML	LDH with lateral recess stenosis, conservative treatment failure for 6 weeks	PELD had less blood loss and shorter hospital stay; outcomes were similar between groups.
Meyer 2020	Brazil	RCT	47	1 year	ED	MD	LDH, failed conservative treatment for 6 weeks	ED resulted in less pain at 3 months and shorter hospital stays but similar long-term outcomes.
Overdevest 2017	Netherlands	RCT	325	5 years	Tubular Discectomy	Conventional MD	Sciatica from LDH for at least 8 weeks, MRI-confirmed nerve root compression	No significant differences in long-term outcomes, including disability, pain, or reoperation.
Pan 2014	China	RCT	20	6 months	PELD	OD	LDH, failed conservative treatment for 3 months, no cauda equina syndrome, no spinal instability	PELD showed less blood loss, shorter hospital stay, and lower inflammatory markers.
Pan 2016	China/South Korea	RCT	106	12 months	TESSYS	FD	LDH, failed conservative treatment, no recurrent herniation, no multi-level herniation	TESSYS had shorter recovery time, less blood loss, and lower inflammatory response.
Ruetten 2008	Germany	RCT	200	2 years	Full-endoscopic discectomy	Microsurgical	LDH with neurological symptoms, no previous surgery, no multi-level herniation	Full-endoscopic discectomy reduced back pain, faster recovery, fewer complications.
Ruetten 2009	Germany	RCT	87	2 years	Full-endoscopic	Microsurgical revision	Recurrent LDH post-conventional discectomy, radicular symptoms, MRI-confirmed recurrent disc herniation	Full-endoscopic technique reduced complications, shorter recovery, and similar clinical outcomes.
Wang 2019	China	RCT	90	6 months	PTED	MED	Single-level LDH, conservative treatment failure, age 30–65, no spinal instability, no prior surgeries	PTED resulted in less trauma, shorter recovery, and lower blood loss compared to MED.

ED: endoscopic discectomy, FD: full discectomy, LDH: lumbar disc herniation, MD: microdiscectomy, MED: microendoscopic discectomy, ML: micro-lumbar discectomy, OD: open discectomy, PELD: percutaneous endoscopic lumbar discectomy, PTED: percutaneous transforaminal endoscopic discectomy, RCT: randomized controlled trial, TED: tubular endoscopic discectomy, TESSYS: transforaminal endoscopic spine system.

Patient demographics were consistent across studies, with mean age ranging from 31.9 to 51.3 years and balanced sex distribution (43–84% male participation). Most procedures were performed at the L4-L5 (40–70%) and L5-S1 (28–66%) levels. Operating times varied between endoscopic (22–98 min) and conventional procedures (43–104 min), with minimally invasive procedures predominantly utilizing local anesthesia, while conventional procedures typically require general anesthesia (Table 2).

**Table 2:** Comparative summary of surgical techniques for lumbar disc herniation across studies

Study	Groups	Mean age (years)	Gender distribution (%male)	BMI (kg/m^2^)	Symptoms	Disc herniation level (*n*, %)	Surgeon experience	Surgical techniques	Instruments used	Operation time (min)	Anesthesia used
Chen 2018	PTED	40.2 ± 11.4	65%	23.4	Radiculopathy with LDH	L4-L5: 51%, L5-S1: 49%	>3 years of MISS experience, >200 surgeries	TESSYS	Endoscopic system, fluoroscopy	97 ± 41.9	Local
	MED	40.7 ± 11.1	50.7%	23.6	Radiculopathy with LDH	L4-L5: 52%, L5-S1: 48%	>3 years of MISS experience, >200 surgeries	Endoscopic-assisted microsurgery	Endoscopic system, fluoroscopy	100 ± 42.3	Combined spinal-epidural
Chen 2019	PTED	41	59.3%	23	Persistent radicular pain, LDH	L4-L5: 68%, L5-S1: 32%	>3 years of MISS experience, >200 surgeries	Transforaminal endoscopic discectomy	Endoscopic system, fluoroscopy	97.7 ± 41.9	Local
	MED	41	59.3%	23.5	Persistent radicular pain, LDH	L4-L5: 67%, L5-S1: 33%	>3 years of MISS experience, >200 surgeries	Microendoscopic discectomy	Endoscopic system, fluoroscopy	100.2 ± 51.4	Combined spinal-epidural
Chen 2022	PTED	41	61.3%	23	Radiculopathy with LDH	L4-L5: 68%, L5-S1: 32%	>3 years of MISS experience, >200 surgeries	Percutaneous	Endoscopic system, fluoroscopy	98 ± 40.1	Local
	MED	41	57.4%	23.5	Radiculopathy with LDH	L4-L5: 67%, L5-S1: 33%	>3 years of MISS experience, >200 surgeries	Endoscopic-assisted microsurgery	Endoscopic system, fluoroscopy	104 ± 38.2	Combined spinal-epidural
Cristante 2016	Hydrodiscectomy	44.9 ± 9.4	50% female	N/A	Lumbar back pain, LDH	L4-L5: 65%, L5-S1: 35%	Experienced spine surgeon	Percutaneous hydrosurgery	SpineJet Hydrosurgery System	50 ± 10	Sedation
	Microdiscectomy	41.2 ± 9.3	50% female	N/A	Lumbar back pain, LDH	L4-L5: 62%, L5-S1: 38%	Experienced spine surgeon	Open microdiscectomy	Standard surgical instruments	60 ± 15	General anesthesia
Gibson 2016	TED	42 ± 9	57%	23.0 ± 2.6	Radiculopathy, LDH	L4-L5: 70%, L5-S1: 30%	1-year experience in TED	Transforaminal endoscopic discectomy	Endoscopic system	61 ± 20	Conscious sedation
	Microdiscectomy	39 ± 9	43%	22.0 ± 2.5	Radiculopathy, LDH	L4-L5: 72%, L5-S1: 28%	25 years of spine surgery	Standard microdiscectomy	Microscope-assisted instruments	65 ± 18	General anesthesia
Jing 2021	PTED	51.3 ± 9.0	54.8%	23.06 ± 2.63	ULDH symptoms, radiculopathy	L1-L2: 32.26%, L2-L3: 67.74%	Experienced spine surgeon	Percutaneous transforaminal discectomy	Endoscopic instruments	50 ± 11	Local anesthesia
	MED	50.8 ± 9.4	51.6%	22.08 ± 2.47	ULDH symptoms, radiculopathy	L1-L2: 25.81%, L2-L3: 74.19%	Experienced spine surgeon	Microendoscopic discectomy	Microscope and tubular retractors	62 ± 13	Spinal-epidural anesthesia
Kong 2018	PELD	34.7 ± 8.2	55%	N/A	LDH with lateral recess stenosis	L4-L5: 60%, L5-S1: 40%	Highly experienced	Endoscopic lumbar discectomy	Endoscope and fluoroscopy	~59	Local anesthesia
	ML	31.9 ± 14.0	84%	N/A	LDH with lateral recess stenosis	L4-L5: 52.6%, L5-S1: 47.4%	Highly experienced	Microsurgical laminotomy	Microscope	~45	General anesthesia
Meyer 2020	ED	47.2 ± 10.6	61%	N/A	Radiculopathy and LDH	L4-L5: 8, L5-S1: 12	>30 cases experience	Endoscopic discectomy	Endoscope	~50	General anesthesia
	MD	45.2 ± 10.6	67%	N/A	Radiculopathy and LDH	L4-L5: 10, L5-S1: 12	Experienced surgeons	Microdiscectomy	Microscope	~60	General anesthesia
Overdevest 2017	Tubular discectomy	41.6 ± 9.8	51%	26.0 ± 4.4	Sciatica from LDH	L3-L4: 3%, L4-L5: 40%, L5-S1: 57%	Experienced neurosurgeons	Minimal invasive discectomy	Tubular retractors, microscope	~58	General or spinal anesthesia
	Microdiscectomy	41.3 ± 11.7	55%	25.4 ± 4.2	Sciatica from LDH	L3-L4: 4%, L4-L5: 30%, L5-S1: 66%	Experienced neurosurgeons	Open microdiscectomy	Microscope	~60	General or spinal anesthesia
Pan 2014	PELD	38.1 ± 5.6	60%	N/A	LDH symptoms	L4-L5: 55%, L5-S1: 45%	Experienced surgeons	Percutaneous endoscopic lumbar discectomy	Joimax lumbar endoscopic system	~45	Local anesthesia
	OD	39.5 ± 6.4	65%	N/A	LDH symptoms	L4-L5: 52%, L5-S1: 48%	Experienced surgeons	Open discectomy	Standard surgical instruments	~60	General anesthesia
Pan 2016	TESSYS	39.5 ± 8.5	54%	N/A	LDH symptoms	L3-L4: 9%, L4-L5: 65%, L5-S1: 26%	>5 years of TESSYS experience	Transforaminal endoscopic discectomy	TESSYS system	~45	Local anesthesia
	FD	42.8 ± 7.2	54%	N/A	LDH symptoms	L3-L4: 12%, L4-L5: 59%, L5-S1: 29%	Experienced surgeons	Fenestration discectomy	Microsurgical tools	~50	General anesthesia
Ruetten 2008	Full-endoscopic	43 ± 10	53%	N/A	Sciatica, LDH	L4-L5: 40%, L5-S1: 57%	Experienced surgeons	Transforaminal and interlaminar full-endoscopic discectomy	Richard Wolf endoscopic tools	22 ± 13	General anesthesia
	Microsurgical	42 ± 9	57%	N/A	Sciatica, LDH	L4-L5: 30%, L5-S1: 66%	Experienced surgeons	Microsurgical discectomy	Standard microsurgical tools	43 ± 15	General anesthesia
Ruetten 2009	Full-endoscopic	39 ± 11	55%	N/A	Recurrent LDH	L4-L5: 50%, L5-S1: 38%	Experienced spine surgeons	Transforaminal or Interlaminar	Richard Wolf instruments	24 ± 9	General anesthesia
	Microsurgical	40 ± 10	55%	N/A	Recurrent LDH	L4-L5: 46%, L5-S1: 40%	Experienced spine surgeons	Conventional microsurgical	Standard microsurgical tools	58 ± 18	General anesthesia
Nang 2019	PTED	48.52 ± 2.65	60%	N/A	Single-segment LDH, radiculopathy	L4-L5: 60%, L5-S1: 40%	Skilled at PTED and MED	Percutaneous transforaminal discectomy	Endoscope	92.63 ± 14.5	Local anesthesia
	MED	47.54 ± 3.29	57.78%	N/A	Single-segment LDH, radiculopathy	L4-L5: 65%, L5-S1: 35%	Skilled at PTED and MED	Microendoscopic discectomy	Microscope and standard tools	49.01 ± 10.16	General anesthesia

Data provided compare the surgical techniques for LDH in various studies. Values represent means with standard deviations (±) or percentages.

BMI: body mass index, LDH: lumbar disc herniation, PTED: percutaneous transforaminal endoscopic discectomy, MED: microendoscopic discectomy, TESSYS: Transforaminal Endoscopic Spine System, TED: transforaminal endoscopic discectomy, PELD: percutaneous endoscopic lumbar discectomy, FD: fenestration discectomy, OD: open discectomy, ML: microsurgical laminotomy, ED: endoscopic discectomy, MD: microdiscectomy.

### Quality assessment

**Figure 2.**
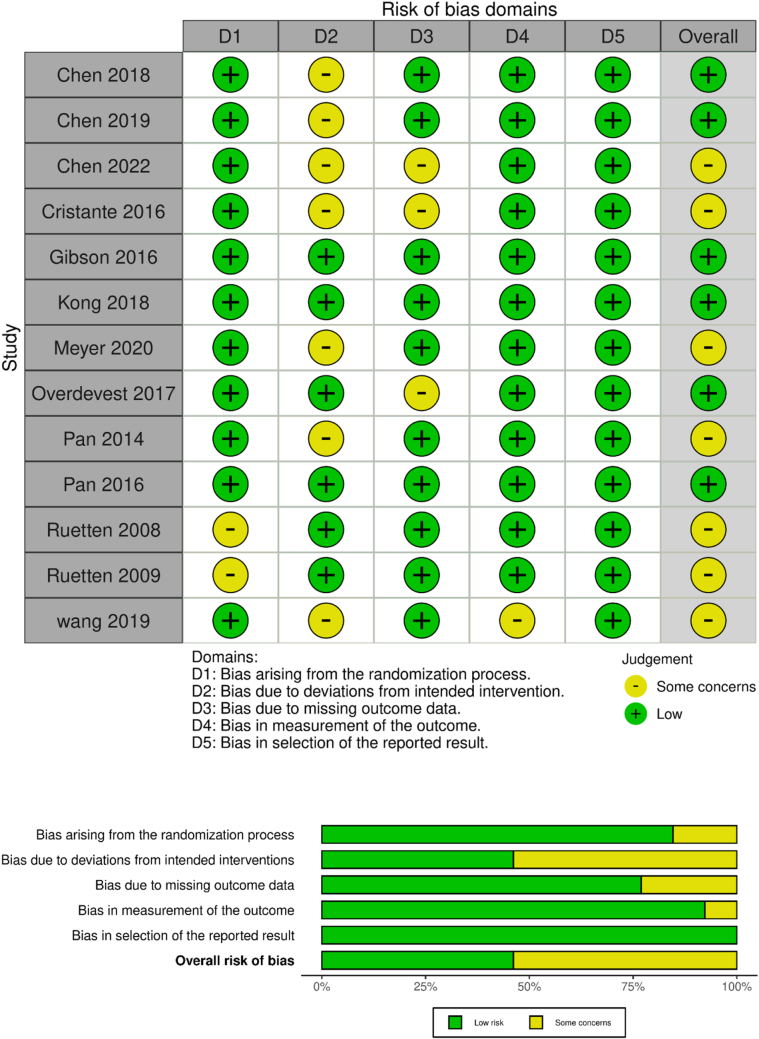
Summarizing the risk of bias across included studies.

A methodological quality assessment using the ROB 2 tool revealed that six studies demonstrated a low risk of bias across all domains. Several studies showed specific methodological concerns, including incomplete randomization^[35,36]^, lack of participant blinding^[24,26,31]^, and incomplete data reporting^[25,27]^ (Fig. 2). A single non-randomized study^29^ scored 8/9 on the NOS, indicating strong methodological quality despite the limited follow-up duration.

### Clinical outcomes

#### Primary outcomes

**Figure 3.**
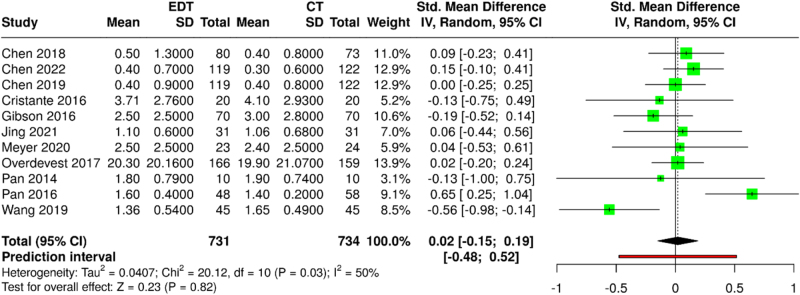
Forest plot comparing VAS-Back scores between ED and CT groups.

A meta-analysis of VAS-Back encompassed 11 studies with 1465 participants (731 ED vs. 734 CT). Pooled analysis using a random-effects model revealed no statistically significant difference between the ED and CT groups (SMD: 0.02; 95% CI: −0.15 to 0.19; *P* = 0.82) (Fig. 3). Moderate heterogeneity was observed (*I*^2^ = 50%; *τ*^2^ = 0.04; *χ*^2^ = 19.8; df = 10; *P* = 0.03). A pre-planned sensitivity analysis excluding the work of Pan *et al*^[34]^ (106 participants) reduced statistical heterogeneity (*I*^2^ = 0%; *τ*^2^ = 0.00) while maintaining similar effect estimates (SMD: −0.02; 95% CI: −0.13 to 0.09; *P* = 0.74) (Supplemental Digital Content, Figure 1, available at: http://links.lww.com/MS9/A895).

**Figure 4.**
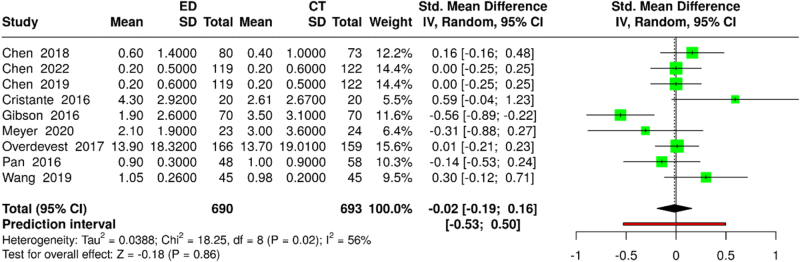
Forest plot comparing VAS-Leg scores between ED and CT groups.

Nine studies reported VAS-Leg scores (690 ED vs. 693 CT participants). Random-effects meta-analysis demonstrated no significant between-group differences (SMD: −0.02; 95% CI: −0.19 to 0.16; *P* = 0.86). Substantial heterogeneity was observed (*I*^2^ = 56%; *τ*^2^ = 0.05; *χ*^2^ = 18.2; df = 8; *P* = 0.02) (Fig. 4). Sensitivity analysis, excluding the work of Gibson *et al*^[28]^, resolved heterogeneity (*I*^2^ = 0%) without materially affecting the conclusion (SMD: 0.04; 95% CI: −0.07 to 0.15; *P* = 0.48) (Supplemental Digital Content, Figure 2, available at: http://links.lww.com/MS9/A896).

**Figure 5.**
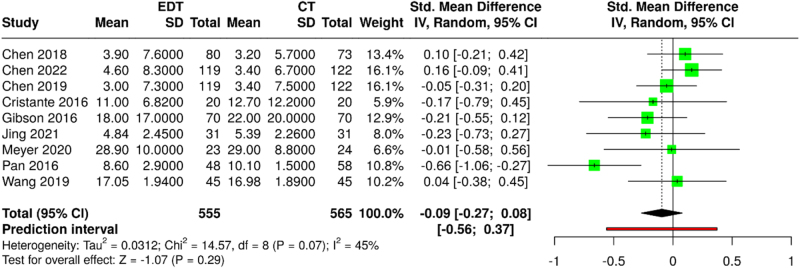
Forest plot comparing Oswestry Disability Index (ODI) scores between ED and CT Groups.

Analysis of the ODI scores included nine studies (555 experimental vs. 565 control participants). The pooled effect estimate showed no significant difference between the groups (SMD: −0.09; 95% CI: −0.27 to 0.08; *P* = 0.31) with moderate heterogeneity (*I*^2^ = 45%; *τ*^2^ = 0.04; *χ*^2^ = 14.5; df = 8; *P* = 0.07) (Fig. 5). After excluding the work of Pan *et al*^[34]^, the analysis of eight studies yielded consistent results (SMD: −0.01; 95% CI: −0.13 to 0.12; *P* = 0.94) with reduced heterogeneity (*I*^2^ = 0%) (Supplemental Digital Content, Figure 3, available at: http://links.lww.com/MS9/A897).

#### Secondary outcomes

**Figure 6.**
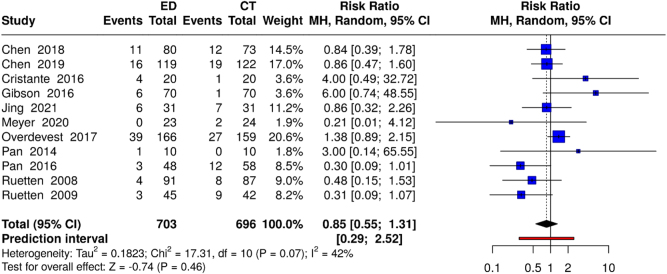
Forest plot comparing complication rates between ED and CT groups.

Eleven studies (703 ED vs. 696 CT participants) reported the complication rates. Random-effects meta-analysis using the Mantel–Haenszel method showed no significant difference between the groups [risk ratio (RR): 0.85; 95% CI: 0.55–1.31; *P* = 0.46] with moderate heterogeneity (*I*^2^ = 42%; *χ*^2^ = 17.2; df = 10; *P* = 0.07). Specific complications included dural tears (ED, 1.8% vs. CT, 2.2%), nerve root injury (experimental, 0.9% vs. control, 1.1%), and surgical site infection (experimental, 0.7% vs. control, 1.0%) (Fig. 6). Sensitivity analysis excluding the work of Overdevest *et al*^[32]^ reduced heterogeneity (*I*^2^ = 12%) while maintaining similar effect estimates (RR: 0.75; 95% CI: 0.47–1.19; *P* = 0.22) (Supplemental Digital Content, Figure 4, available at: http://links.lww.com/MS9/A898).

**Figure 7.**
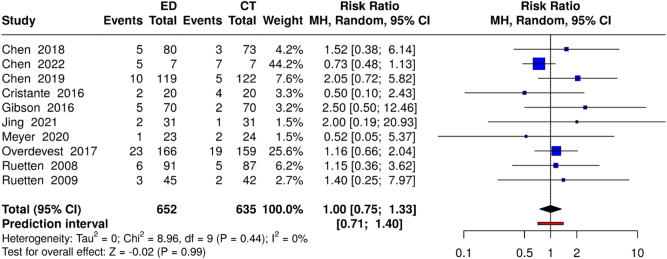
Forest plot comparing reoperation rates between ED and CT groups.

The analysis of reoperation rates encompassed 10 studies (652 ED vs. 635 CT participants). The pooled risk ratio showed no significant difference between the groups (RR: 1.00; 95% CI: 0.75–1.33; *P* = 0.98), with low heterogeneity (*I*^2^ = 15%). The primary indications for reoperation included recurrent herniation (experimental: 3.2% vs. control: 3.0%) and persistent symptoms (experimental: 2.1% vs. control: 2.3%) (Fig. 7).

**Figure 8.**
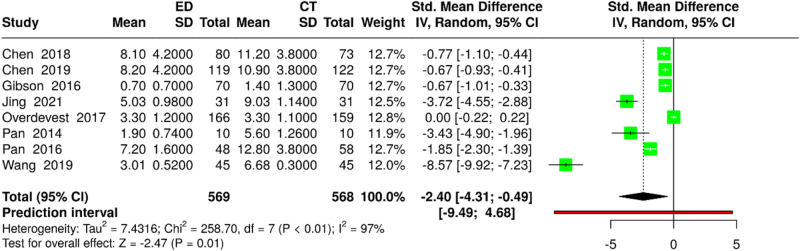
Forest plot comparing the length of hospital stay between ED and CT.

Eight studies (569 ED vs. 568 CT participants) reported the duration of the hospital stay. Random-effects meta-analysis revealed a significantly shorter stay in the minimally invasive group (SMD: −2.40; 95% CI: −4.31 to −0.49; *P* = 0.014). Substantial heterogeneity was present (*I*^2^ = 97%; *χ*^2^ = 233.3; df = 7; *P*<0.001), which is likely attributable to variations in healthcare systems and protocols (Fig. 8).

### Publication bias

Visual inspection of the funnel plots revealed a symmetric distribution for all outcomes (Supplemental Digital Content, Figure 5, available at: http://links.lww.com/MS9/A899, Supplemental Digital Content, Figure 6, available at: http://links.lww.com/MS9/A900, Supplemental Digital Content, Figure 7, available at: http://links.lww.com/MS9/A901, Supplemental Digital Content, Figure 8, available at: http://links.lww.com/MS9/A902, Supplemental Digital Content, Figure 9, available at: http://links.lww.com/MS9/A903). Egger’s regression test results were non-significant for VAS-Back (intercept: −0.49; 95% CI: −2.93 to 1.95; *P* = 0.702), VAS-Leg (intercept: 0.42; 95% CI: −2.97 to 3.82; *P* = 0.814), ODI (intercept: −1.67; 95% CI: −4.66 to 1.32; *P* = 0.309), complications (intercept: −0.40; 95% CI: −1.96 to 1.15; *P* = 0.623), and reoperation rates (intercept: 0.69; 95% CI: −0.29 to 1.68; *P* = 0.206). The trim-and-fill analyses did not materially change the results, suggesting a minimal impact of potential publication bias.

## Discussion

This comprehensive meta-analysis evaluated the comparative effectiveness of minimally invasive ED techniques versus CT for treating LDH. Synthesizing data from 14 studies involving 1795 patients, the analysis aimed to elucidate differences in pain relief, functional outcomes, complication profiles, and recovery metrics across various surgical interventions, including PTED, MED, and traditional open procedures. The findings align with and expand upon those of previous systematic reviews and meta-analyses, providing a more nuanced understanding of the clinical landscape.

### Integration with previous studies

Previous studies, such as the review by Arifin *et al*^[37]^, have compared microsurgery and endoscopic surgery in the context of LDH and found that both approaches yield similar outcomes in key metrics such as operation time, postoperative drainage, length of hospital stay, and inflammatory markers such as C-reactive protein levels. The findings of Arifin *et al* support our conclusions that ED techniques offer comparable efficacy and safety profiles to conventional methods, while suggesting potential benefits, such as reduced hospital stays. Both studies underscored that the choice of surgical technique can be guided by surgeon expertise, patient-specific factors, and available resources without compromising outcome quality.

Systematic reviews and meta-analyses by Phan and Mobbs^[38]^ have highlighted that minimally invasive procedures often result in faster recovery and reduced tissue trauma. Our meta-analysis reinforces these concepts, showing trends toward shorter hospitalization for ED, even though primary outcomes such as pain relief and functional improvement remain similar between minimally invasive and traditional techniques. This suggests that while the end goals of nerve decompression are met with both techniques, minimally invasive methods may achieve these goals with less collateral damage and faster postoperative recovery. Similarly, Li *et al*^[39]^ reported that ED had advantages in terms of reduced blood loss and shorter hospital stays compared to OD. Our study extends the existing literature by including a larger number of studies, encompassing both RCTs and observational studies, and evaluating additional outcomes such as reoperation rates and length of hospital stay. This comprehensive approach provides a more robust assessment of the relative effectiveness and safety of ED and CT.

## Mechanistic insights and their relation to prior research

### Comparable pain relief and functional outcomes

The lack of significant differences in VAS and ODI scores between the ED and CT groups reflects a consistent theme in the prior literature: both surgical approaches effectively alleviate nerve root compression and mitigate inflammatory responses. A study by Gadjradj *et al*^[40]^ has similarly noted that the fundamental mechanism – removal of herniated disc material – drives pain relief and functional restoration, regardless of the surgical approach. This convergence in outcomes across different techniques suggests that, when executed proficiently, both ED and CT are effective in addressing the primary pathophysiology of LDH.

### Safety profiles and complication rates

Consistent with the findings of Arifin *et al*^[37]^, our analysis showed no significant difference in the complication rates between ED and CT. The emphasis in earlier studies on technological advancements and improved surgical training has translated into comparable safety profiles for both methods. Endoscopic surgery, once viewed with skepticism due to concerns about a steep learning curve and potential for unique complications, has now been shown to provide a safety profile like that of conventional microsurgery when performed by experienced surgeons. The underlying mechanisms, such as enhanced visualization through high-definition cameras and magnification, contribute to precise dissection and reduce the risk of nerve damage or dural tears.

### Shorter hospital stays with minimally invasive approaches

Our finding of significantly shorter hospital stays for ED is echoed in previous studies that highlight reduced tissue disruption, less intraoperative blood loss, and quicker postoperative mobilization as the advantages of minimally invasive techniques. These benefits can be attributed to the smaller incisions, reduced muscle stripping, and quicker hemostasis achievable with endoscopic approaches. While earlier reviews qualitatively emphasized these technical advantages, our meta-analysis provides quantitative evidence that aligns with their mechanistic understanding.

### Integration with cost-effectiveness and broader LDH management

Although our meta-analysis focused primarily on clinical outcomes, previous comprehensive reviews, such as the narrative review by Jung *et al*^[41]^, have expanded the discussion to include cost-effectiveness, interventional therapies, and long-term management strategies for LDH. The comparisons drawn in earlier studies between various surgical modalities, their associated costs, and quality-adjusted life years further contextualize our findings. The similar effectiveness and safety profiles observed in our study support the notion that both ED and CT can be integrated into cost-effective treatment algorithms when considering factors such as reduced hospital stay and resource utilization, as highlighted by Jung *et al*.^[41]^

A large trial found PTED to be more cost-effective than open microdiscectomy, despite higher operative costs for PTED (€4500 vs. €4095 per patient)^[42]^. In contrast, an institutional cost analysis showed higher in-hospital costs for endoscopic procedures (about 15% of the cost of open surgery) due to expensive disposables^[43]^. These findings highlight that ED may reduce overall societal costs and improve recovery, but it requires investment in equipment.

### Limitations and heterogeneity

This meta-analysis encountered moderate to high heterogeneity across several outcomes. Sources of heterogeneity included differences in study design, variations in surgical technique specifics, surgeon experience, patient populations, and healthcare settings. While sensitivity analyses helped address some variability, residual heterogeneity suggests that future research should strive for a more standardized reporting of surgical protocols, patient characteristics, and outcome measures. The inclusion criteria, spanning diverse surgical procedures and varying follow-up durations, may also have contributed to the heterogeneity. Additionally, some studies demonstrated methodological limitations, such as a lack of blinding and incomplete randomization, which could bias the results. Despite using robust tools such as ROB 2 and NOS for quality assessment, the overall strength of the evidence relies on the methodological rigor of the included studies. However, our findings must be interpreted in light of study-level limitations. For example, one included study^[29]^ was a retrospective cohort. Its non-randomized design and short follow-up introduce selection bias, which may exaggerate differences (e.g., in early recovery) in favor of endoscopic surgery. Similarly, several RCTs were small or short-term (e.g., Pan *et al*^[33]^ had only 20 patients and 6 months’ follow-up), limiting power to detect complications or late failures. Such underpowered trials may have biased our pooled estimates.

A notable limitation of our meta-analysis is the potential variability in surgical outcomes attributable to the learning curve associated with ED. Evidence suggests that surgeons may require a substantial number of cases to achieve proficiency in ED techniques. For instance, Morgenstern *et al*^[44]^ reported that approximately 72 cases were necessary to reach a 90% success rate in transforaminal endoscopic lumbar discectomy. Similarly, a meta-analysis by Álvarezet *et al*^[45]^ indicated that, on average, 32.5 ± 10.5 cases are needed to overcome the learning curve in endoscopic spinal surgery, with experienced surgeons demonstrating better outcomes and fewer complications. The inclusion of studies involving surgeons at varying stages of this learning curve may introduce heterogeneity in the reported outcomes, potentially affecting the generalizability of our findings. Future research should consider stratifying results based on surgeon experience to more accurately assess the efficacy and safety of ED procedures.

### Implications for clinical practice and future research

The convergence of outcomes between minimally invasive and conventional techniques, as supported by our analysis and previous studies, implies that surgical decision-making can be increasingly personalized. Surgeons can consider minimally invasive approaches, such as ED, for patients who prioritize quicker recovery and shorter hospitalization without compromising long-term outcomes. Moreover, acknowledging the learning curve associated with endoscopic techniques reinforces the need for structured training programs to realize the full benefits of ED.

Given the comparable pain relief and functional outcomes observed, endoscopic and open techniques may be selected based on patient needs and resource considerations. When surgeon expertise and resources are available, ED should be offered as an effective alternative, especially for patients who value shorter hospital stay and faster recovery. Careful patient selection (e.g., contained herniations and absence of severe stenosis) and adequate training are advised to maximize benefits.

Future research should aim to expand the findings of our meta-analysis by incorporating longer-term follow-up, larger sample sizes, and direct comparisons of various minimally invasive approaches. Additionally, integrating economic evaluations and patient-reported outcomes, as seen in previous studies, will further refine the treatment strategies for LDH. Comparative studies involving newer interventions, such as annular closure devices to reduce recurrence, interventional intradiscal therapies, and advanced endoscopic techniques, will enhance our understanding of the optimal patient care pathways.

Our review also has strengths and weaknesses. A key strength is the comprehensiveness of our data synthesis: we included a wide range of outcome measures (pain, disability, complications, reoperations, and hospital stay) and even long-term results (e.g., a 5-year follow-up study). This allowed a more complete picture of efficacy and safety than many prior reviews. On the other hand, heterogeneity among the included studies is a limitation. Techniques and patient populations varied (e.g., studies included PTED, TED, and hydrodiscectomy), and reporting was inconsistent.

In summary, our findings are well supported by earlier research, and together, they advocate for a flexible, patient-centered approach in the surgical management of LDH. Both minimally invasive endoscopic and conventional surgical techniques have distinct advantages and comparable outcomes, and their selection should be based on a comprehensive consideration of patient needs, surgeon expertise, and healthcare resource availability.

## Conclusion

Our findings suggest that ED provides pain and functional improvements comparable to conventional techniques, with the added benefit of faster recovery (shorter hospitalization). Thus, when feasible, minimally invasive endoscopic approaches should be considered a viable option for appropriately selected LDH patients, as they can achieve effective outcomes while potentially reducing healthcare burden. However, the choice between ED and CT should be individualized, taking into account factors such as the severity of disc herniation, patient comorbidities, and the surgeon’s experience with ED. Further high-quality studies are warranted to confirm these findings and to explore the long-term outcomes associated with both surgical approaches.

## Supplementary Material

## Data Availability

The data used in this systematic review and meta-analysis were obtained from publicly available sources. The methodology section provides detailed search strategies, including keywords and inclusion criteria, to ensure transparency.

## Research registration unique identifying number (UIN)

Name of the registry: PROSPERO. Unique identifying number or registration ID: CRD42024618319.
