# Supplementary figures and images for: Comparative effectiveness of minimally invasive endoscopic discectomy versus conventional surgical techniques for lumbar disc herniation: a systematic review and meta-analysis
Source: Ann Med Surg (Lond). 2025 Aug 11;87(10):6661–74. doi: 10.1097/MS9.0000000000003689 (PMC12577904; doi:10.1097/MS9.0000000000003689)

**
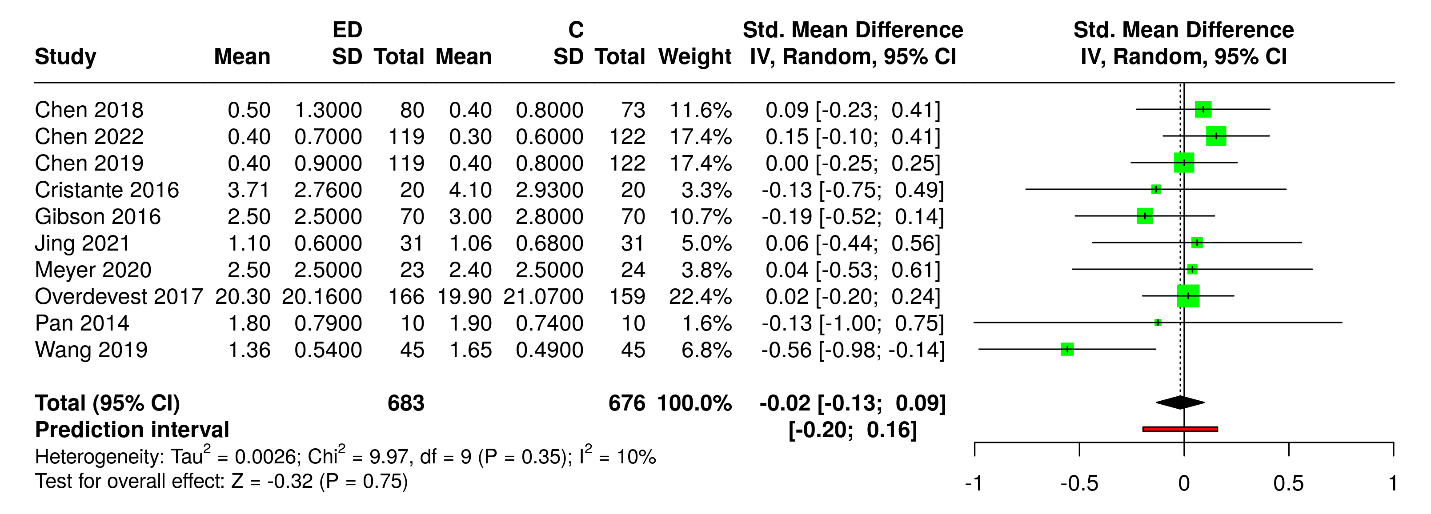
**

**Supplementary Figure 1. Sensitivity analysis for VAS-Back scores excluding Pan 2016.**

Supplement: Supplementary file 1 [file ms9-87-6661-s001.docx]

**
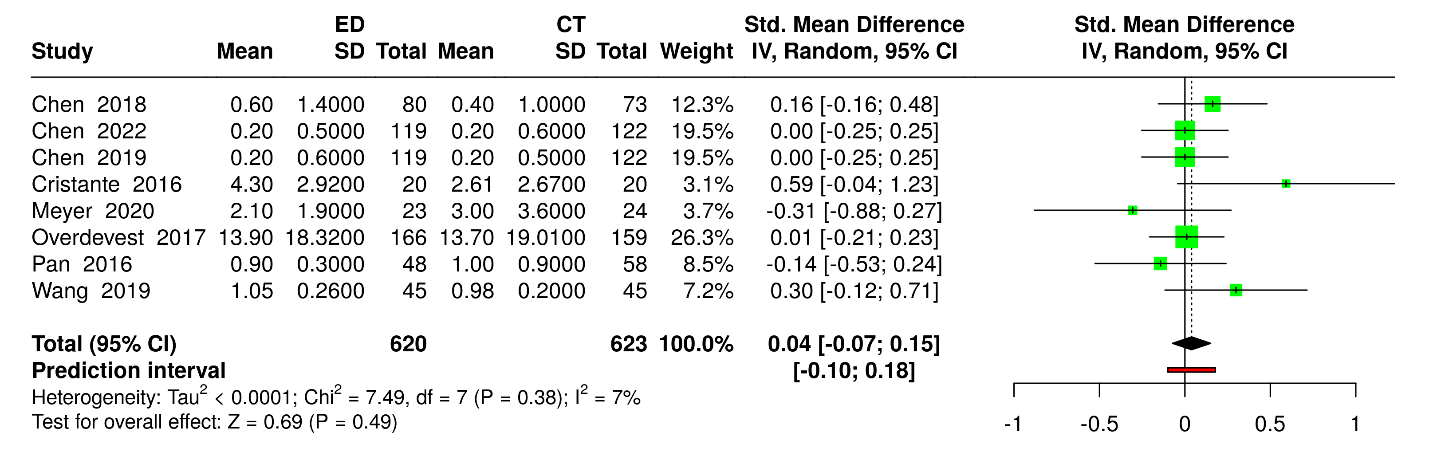
**

**Supplementary Figure 2. Sensitivity analysis for VAS-Leg scores, excluding Gibson (2016)**

Supplement: Supplementary file 2 [file ms9-87-6661-s002.docx]

**
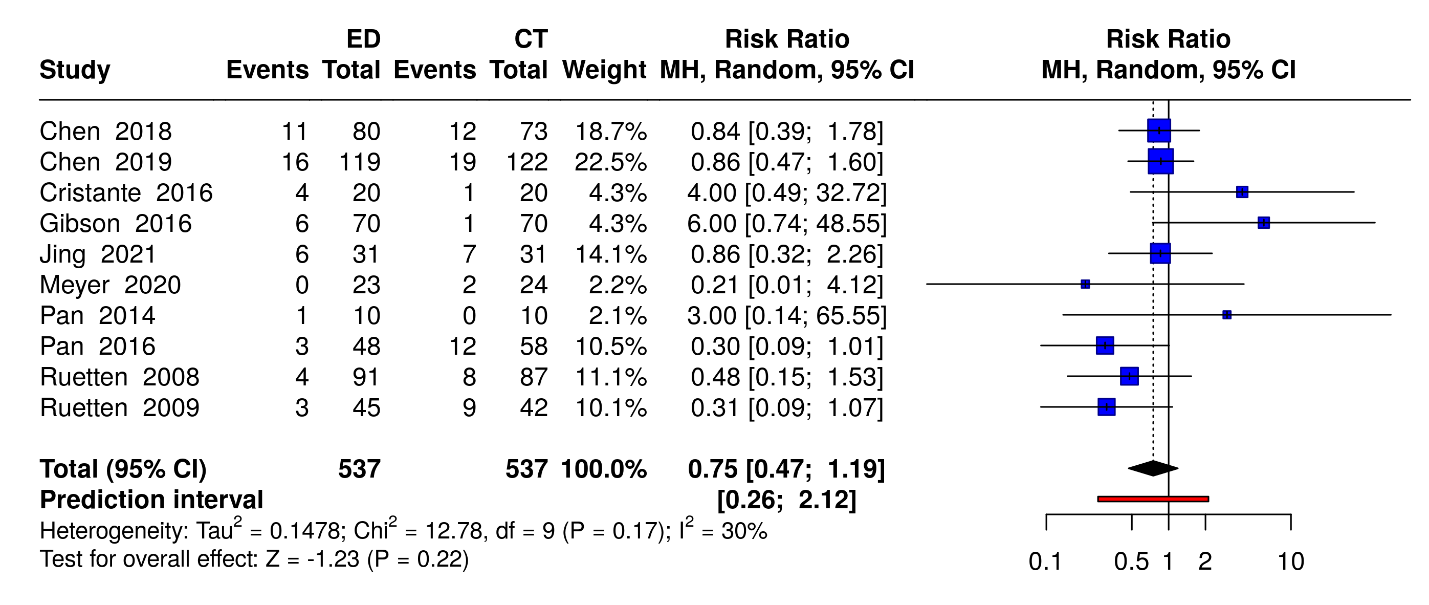
**

**Supplementary Figure 4. Sensitivity analysis for complication rates, excluding Overdevest 2017.**

Supplement: Supplementary file 4 [file ms9-87-6661-s004.docx]

**
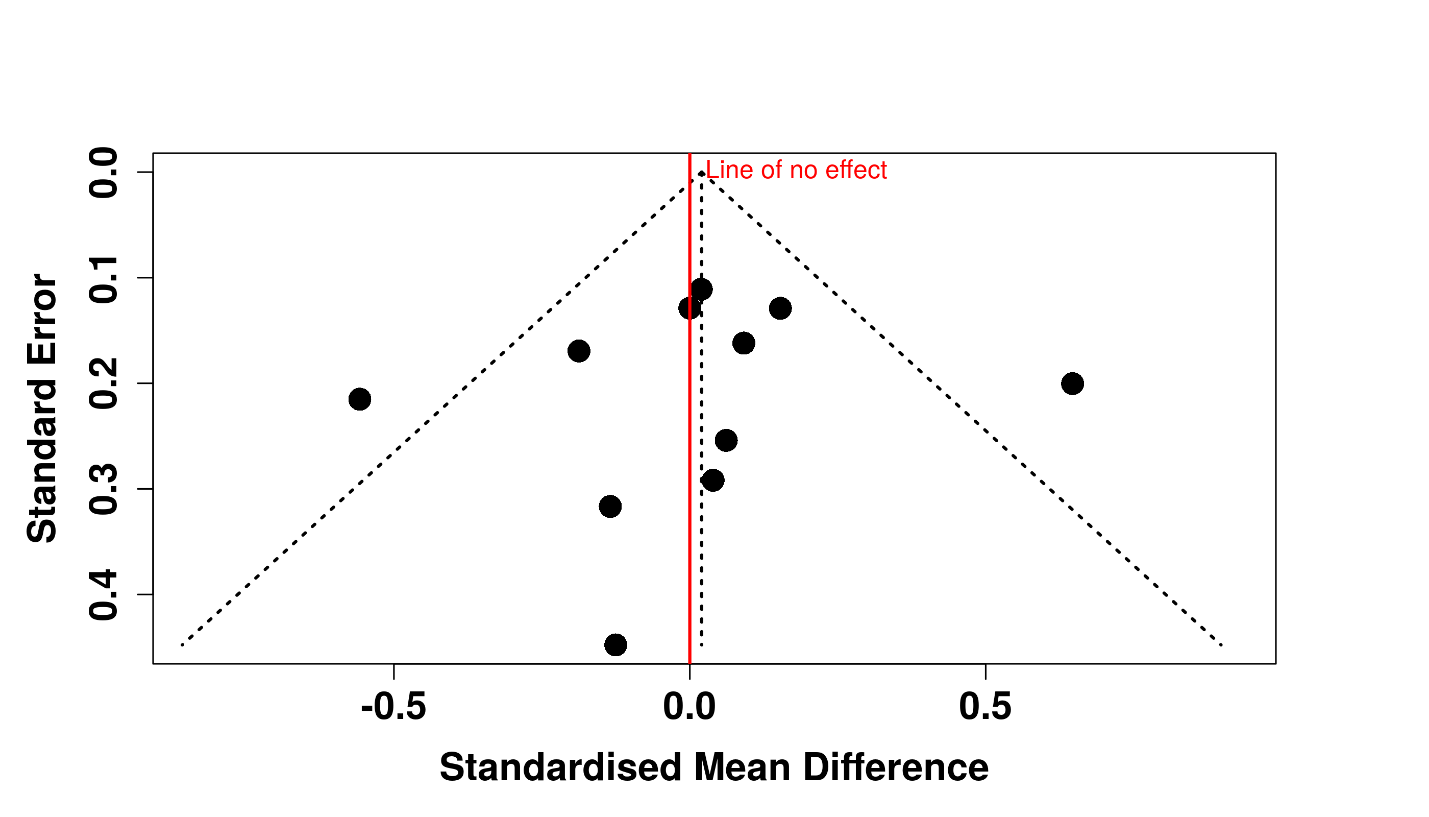
**

**Supplementary Figure 5. Funnel plot assessing publication bias for VAS-Back scores.**

Supplement: Supplementary file 5 [file ms9-87-6661-s005.docx]

**
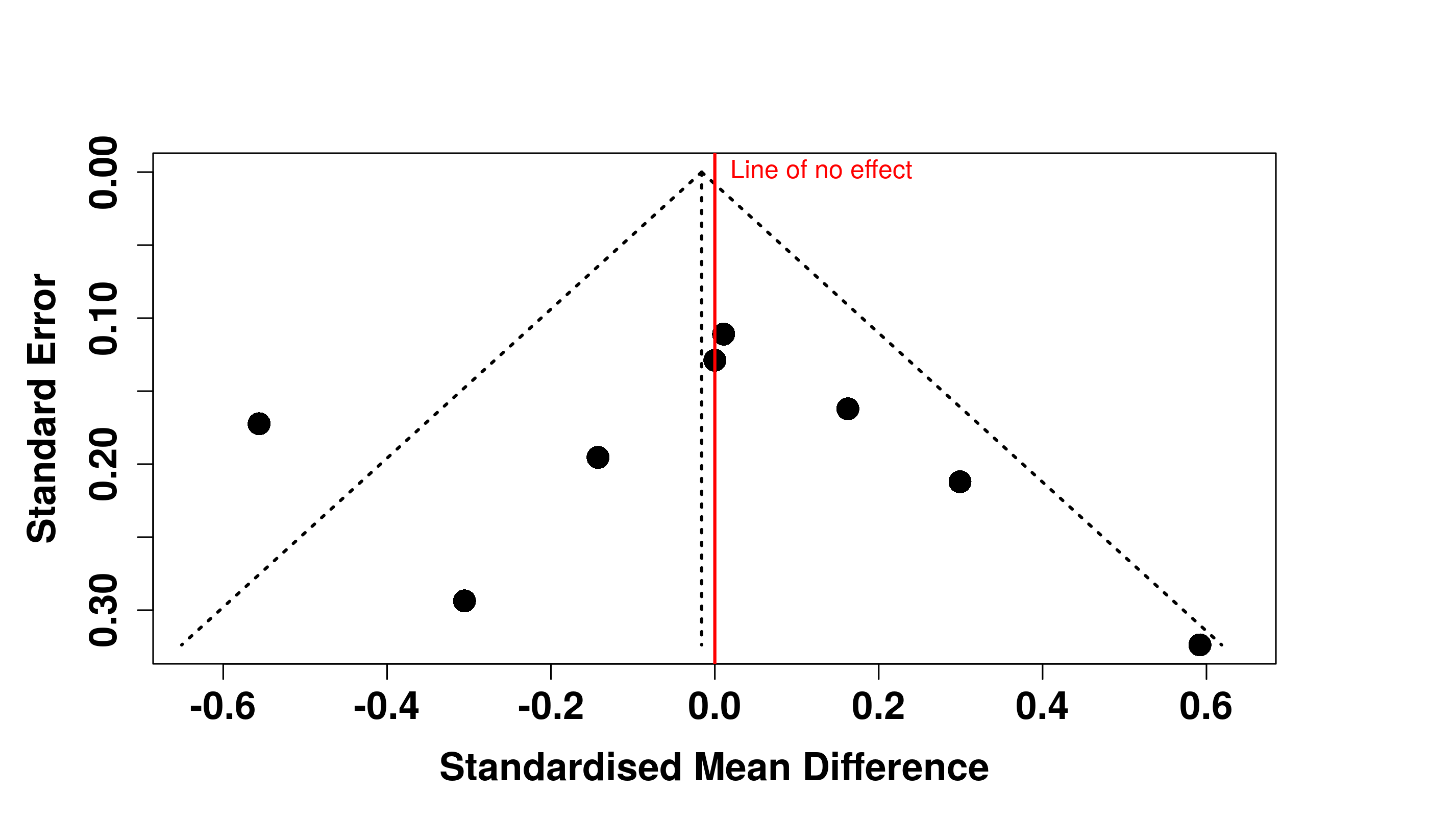
**

**Supplementary Figure 6. Funnel plot assessing publication bias for VAS-Leg scores.**

Supplement: Supplementary file 6 [file ms9-87-6661-s006.docx]

**
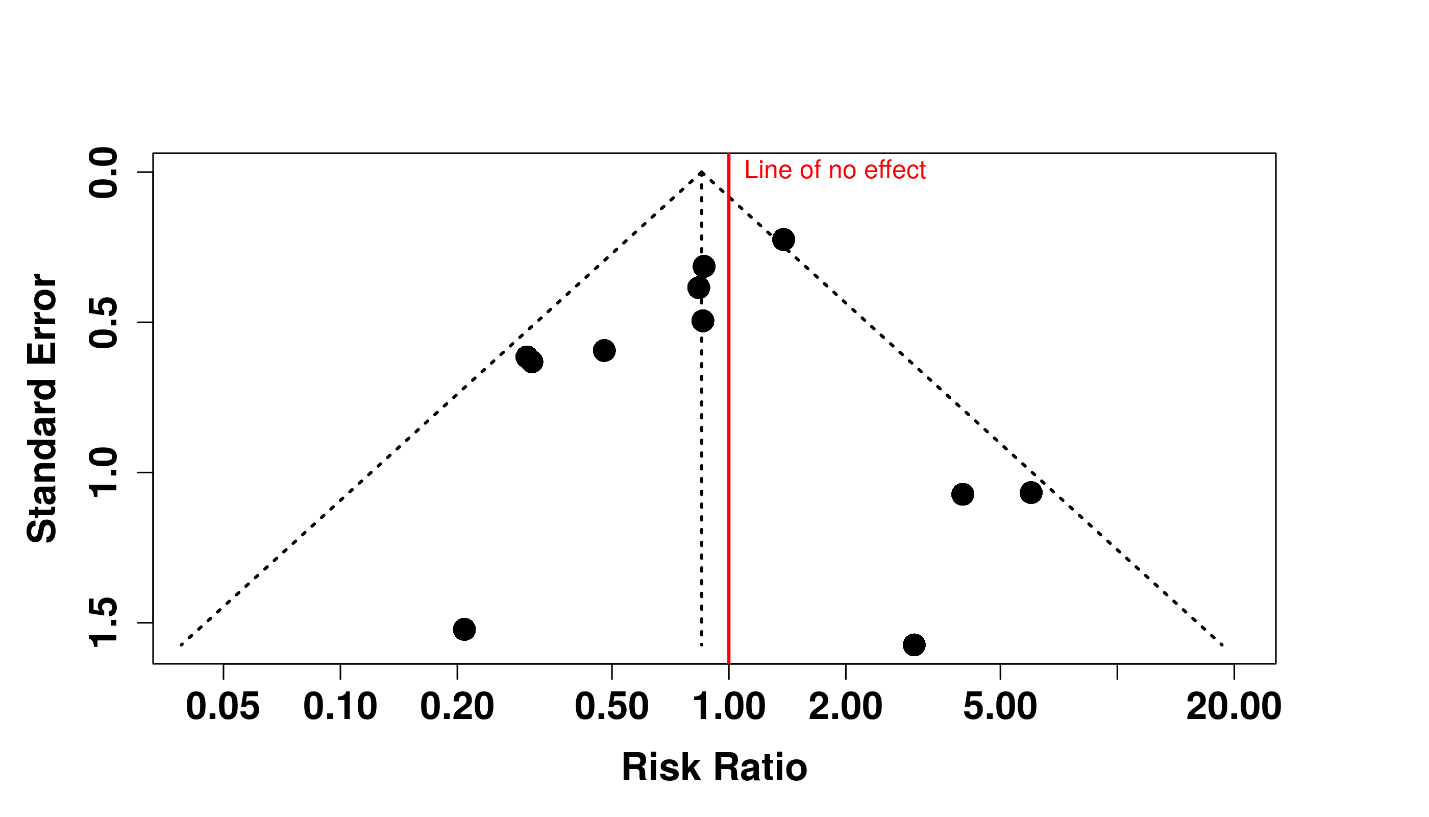
**

**Supplementary Figure 8. Funnel plot assessing publication bias for complication rates.**

Supplement: Supplementary file 8 [file ms9-87-6661-s008.docx]

**
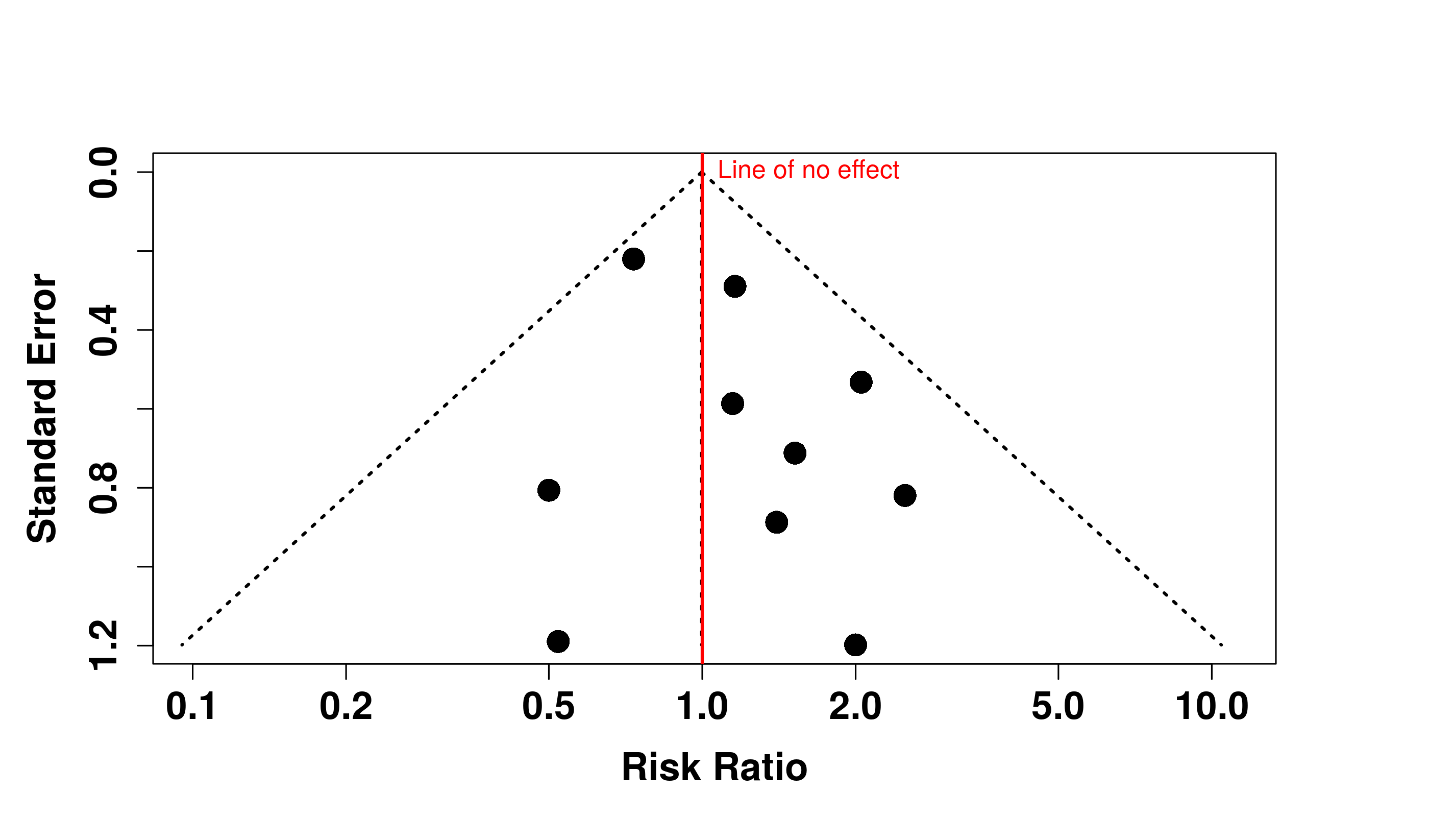
**

**Supplementary Figure 9. Funnel plot assessing publication bias for reoperation rates**.

Supplement: Supplementary file 9 [file ms9-87-6661-s009.docx]
