# Supplementary material for: Comparative effectiveness of minimally invasive endoscopic discectomy versus conventional surgical techniques for lumbar disc herniation: a systematic review and meta-analysis
Source: Ann Med Surg (Lond). 2025 Aug 11;87(10):6661–74. doi: 10.1097/MS9.0000000000003689 (PMC12577904; doi:10.1097/MS9.0000000000003689)
Supplement: Supplementary file 3 [file ms9-87-6661-s003.docx]

**
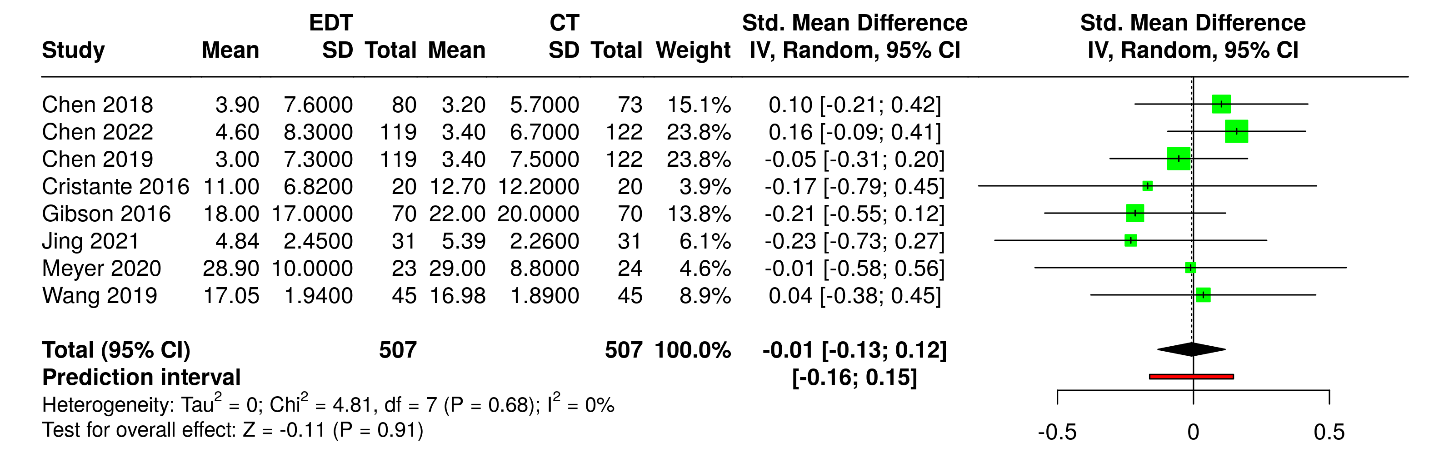
**

**Supplementary Figure 3. Sensitivity analysis for Oswestry Disability Index (ODI) scores excluding Pan 2016.**
